# US28: HCMV’s Swiss Army Knife

**DOI:** 10.3390/v10080445

**Published:** 2018-08-20

**Authors:** Benjamin A. Krishna, William E. Miller, Christine M. O’Connor

**Affiliations:** 1 Genomic Medicine, Lerner Research Institute, Cleveland Clinic, Cleveland, OH 44195, USA; krishnb2@ccf.org; 2 Department of Molecular Genetics, Biochemistry, & Microbiology, University of Cincinnati, Cincinnati, OH 45267, USA; millerwe@ucmail.uc.edu

**Keywords:** cytomegalovirus, *US28*, HCMV

## Abstract

*US28* is one of four G protein coupled receptors (GPCRs) encoded by human cytomegalovirus (HCMV). The US28 protein (pUS28) is a potent signaling molecule that alters a variety of cellular pathways that ultimately alter the host cell environment. This viral GPCR is expressed not only in the context of lytic replication but also during viral latency, highlighting its multifunctional properties. pUS28 is a functional GPCR, and its manipulation of multiple signaling pathways likely impacts HCMV pathogenesis. Herein, we will discuss the impact of pUS28 on both lytic and latent infection, pUS28-mediated signaling and its downstream consequences, and the influence this viral GPCR may have on disease states, including cardiovascular disease and cancer. We will also discuss the potential for and progress towards exploiting pUS28 as a novel therapeutic to combat HCMV.

HCMV is a ubiquitous member of the herpesviridae family, which characteristically establishes lifelong, latent infections that are largely asymptomatic in healthy individuals, as the virus is controlled by a robust immune response. Despite this, HCMV is linked with cardiovascular diseases (CVD) and cancers, where it is potentially an oncomodulatory virus [1,2]. In immuno-compromised, immuno-suppressed, and immuno-naïve patients, CMV can cause devastating disease that often results in mortality. HCMV is the leading cause of congenital birth defects in the US, ahead of fetal alcohol syndrome and far exceeding those caused by Zika virus infection [3]. In immuno-competent children and adults, primary infection with HCMV rarely causes significant health problems. Rather it is primary infection or reactivation of a latent infection in individuals with severely weakened immune systems that pose significant risk. In these cases, HCMV-associated disease is treated with antivirals such as ganciclovir, foscarnet, or letermovir [4], which are effective against lytically replicating virus. However, these treatments are associated with drug toxicity and viral resistance [5,6]. Importantly, latently infected cells are masked from attack by a healthy immune response and are not targets of any current antiviral treatments. Because of this, new therapeutics that target the latent, in addition to the lytic phase, of infection are needed.

The herpesviruses have large DNA genomes, and the beta- and gamma-herpesvirus families encode G protein-coupled receptor (GPCR) homologues [7]. HCMV is no exception, as it encodes four such homologues including *UL33*, *UL78*, *US27*, and *US28*. Three of these viral genes were initially identified by sequence homology analyses of the HCMV genome to the cellular GPCR, rhodopsin [8], while *UL78* was discovered upon comparing the HCMV genome to that of human herpesvirus-6 (HHV-6) [9]. *US28*, the subject of this review, is a well-studied GPCR homologue. Herein, we will review its role as a signaling molecule, its involvement in HCMV models of CVD and cancers, and its requirement for HCMV latent infection.

## 1. Chemokine Receptors

GPCRs are seven-transmembrane signaling proteins that represent a large and diverse group of eukaryotic signaling proteins with a transmembrane structure, which respond to an array of different stimuli acting via a common signaling mechanism [10]. GPCRs interact with heterotrimeric guanine nucleotide-binding regulatory proteins (commonly referred to as G-proteins), comprised of the three subunits termed Gα, Gβ, and Gγ. These heterotrimeric G proteins embedded in the intracellular face of the plasma membrane are bound to guanine diphosphate (GDP) while in their resting state. External cues trigger a conformational change in GPCR structure, which facilitates activation of the Gα subunit, by exchanging the GDP molecule for guanine triphosphate (GTP). This change results in the dissociation of not only the G protein heterotrimers from the GPCR, but also that of the alpha- from the beta-/gamma-subunits. This subsequently leads to their diffusion laterally across the plasma membrane, allowing them to interact with other proteins and activate downstream cellular signaling. GPCR-mediated signaling alters a variety of cellular events, including cell adhesion and migration, mitosis, the regulation of gene expression, and cell–cell communication [11]. As cell surface signaling proteins with a range of different functions, GPCRs are attractive drug targets. Thus, it is no surprise that over 1/3rd of approved therapeutics in the US target the GPCR family [12,13,14].

Chemokine receptors are a subfamily of Class A GPCRs, which bind chemokine proteins to activate cellular signaling, most often causing cellular migration [15]. Chemokines are a type of cytokine, which represent a large family of structurally related, small, intercellular signaling proteins that are predominantly secreted. Best known for their role in inducing chemotaxis, chemokines are divided into four subfamilies based on the positioning of cysteine residues in the N-terminal region: CC, CXC, CX_3_C, and C [16]. These cysteines form structural disulfide bonds that affect the tertiary folding of the protein. The marginal differences in chemokine structure between the four subfamilies mean that most chemokine receptors only bind one subfamily of chemokine, although the majority of chemokine receptors respond to multiple chemokines within one subfamily [15,17].

**Figure 1:**
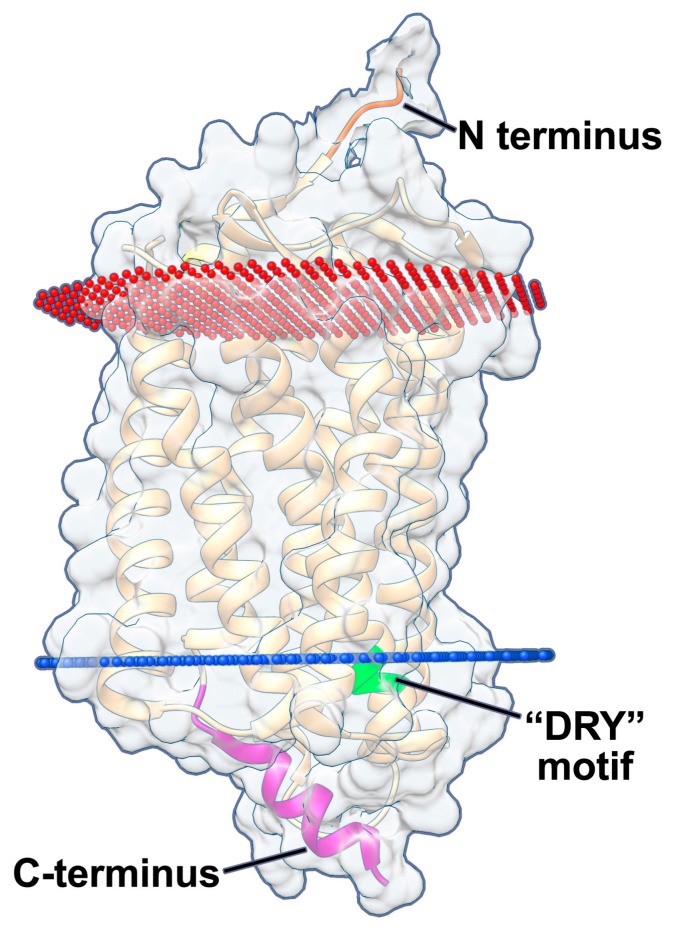
HCMV pUS28 structure. The viral GPCR, pUS28 contains seven transmembrane spanning helices (beige). The N-terminus (orange) of the protein is extracellular and required for ligand binding, while the C-terminal domain (purple) is intracellular and important for protein trafficking. The Y16F ligand binding domain mutant is a single amino acid point mutation at position 16 found within the N-terminus (orange). The canonical “DRY” motif (green) is located within the second intracellular loop and is required for G-protein coupling. The R129A and R129Q mutants discussed herein mutate the arginine (R) of the “DRY” motif to an alanine (A) or glutamine (Q), which ablates G-protein coupling. Extracellular membrane, red; intracellular membrane, blue. The structure is based on the pUS28 crystal structure, bound to CX_3_CL1 ([34]; PDB ID: 4XT3), using Chimera [35] and the Orientations of Proteins in Membranes (OPM) databases [36].

Chemokine receptors have a number of common characteristics in their structures (Figure 1). They require their N-terminus for “hooking” onto chemokines, before pulling the chemokine into the barrel structure by forming multiple low affinity interactions to induce a conformational change in the receptor. The N-terminus is often necessary for chemokine interactions and usually includes both acidic residues as well as a sulfonated tyrosine residue [18,19,20,21]. Ligand binding, and the subsequent interaction of the chemokine with the transmembrane spanning domains, stabilizes an open conformation on the intracellular face of chemokine receptors, exposing a conserved aspartate-arginine-tyrosine (DRY) motif on the second intracellular loop, which interacts with the Gα protein subunit of the heterotrimer. Certain GPCRs can bind multiple types of ligands, which stabilize different open conformations, leading to signaling via distinct G proteins [22,23,24]. Disruption of the conserved DRY motif exhibits different effects for different chemokine receptors, however mutation of the arginine residue in the DRY motif usually ablates signal transduction [25,26,27,28]. Chemokine receptors are also regulated at the C-terminus through phosphorylation of serine and threonine residues, either by GPCR kinases (GRKs) or by second messenger kinases, such as protein kinases A and C (PKA and PKC, respectively) [29]. This phosphorylation leads to binding of arrestin, which desensitizes the receptor, thus preventing further signal transduction and allows subsequent internalization, after which the receptor can either be recycled to the cell surface or degraded [30]. Phosphorylation, as well as arrestin binding, varies in a ligand- and tissue-specific manner [29,31,32,33].

Ligand activation of GPCRs promotes interactions with Gα proteins. These Gα proteins are further divided into subfamilies based on sequence similarity: Gα_i/o_, Gα_q/11_, Gα_12/13_, Gα_s_, Gα_t_, and Gα_olf_ [37]. Members of these distinct subfamilies display different tissue distribution and signaling properties. For example, Gα_i_ and Gα_q_ have wide tissue distribution, while Gα_15_ and Gα_16_ display myeloid and lymphoid lineage specificity [37,38]. This wide range of ligand binding, combined with a wide selection of Gα proteins explains how the structurally similar GPCRs can induce multiple, yet distinct signaling pathways, altering various cellular processes that result in a milieu of phenotypes across the whole of biology.

## 2. Expression of HCMV-Encoded Chemokine Receptor Homologues and Their Interactions

All four HCMV chemokine receptor homologues are expressed during lytic infection [39,40], but the functions of the US28 protein (pUS28) are better defined. This is in part due to the fact that *US27*, *UL33*, and *UL78* encode orphan receptors with no known ligand(s) [41,42]. While pUS28, pUL33, and pUL78 are each dispensable for lytic infection of fibroblasts [40,43,44,45,46], pUS27 is required for infection of these same cells at low multiplicities of infection, where it contributes to extracellular spread of the virus [47]. pUL78 is, however, required for efficient viral replication in both endothelial and epithelial cells and is necessary for entry and virion delivery into epithelial cells [45]. Similar to pUL78, Noriega and colleagues have shown that pUS28 also augments epithelial cell infection [48]. While pUS27 does not appear to have constitutive signaling activity [49], in collaboration with the Spencer Lab, we have recently shown that it potentiates CXCR4-mediated calcium release [42] and enhances CXCR4 activation [50]. To date, there is no evidence that pUL33 can signal during infection, however this viral GPCR does constitutively activate phospholipase C (PLC) in transiently transfected Cos-7 cells [49,51]. Discussed in more detail below, data suggests that *US27* is not expressed during latency [52], however both *UL33* and *UL78* were detected in a recent RNA sequencing (RNA-seq) analyses of viral transcripts from both experimentally and naturally latently infected CD34^+^ hematopoietic progenitor cells (HPCs) [53]. A summary of these data are presented in Table 1.

Many cellular GPCRs form dimeric or oligomeric complexes, which modulate their trafficking and signaling properties [57,58]. Similarly, pUS28 dimerizes with the other HCMV chemokine receptor homologues in overexpression studies using 293T human embryonic kidney (HEK293T) cells. Although no functional changes were observed between the pUS28:pUS27 dimer, the pUS28:pUL33 and pUS28:pUL78 show reduced pUS28-mediated activation of nuclear factor kappa-light-chain-enhancer of activated B cells (NFκB) signaling (ref. [54]; Table 1). HCMV-encoded chemokine receptor homologues also appear to interact with other such receptors. Both pUL33 and pUL78 heteromerize with CCR5 and CXCR4, which negatively impacts the respective signaling functions of these cellular receptors when overexpressed in the monocytic THP-1 cell line (ref. [41]; Table 1) This suggests that perhaps the HCMV-encoded chemokine receptor homologues may have evolved to modulate the activity of each other, or indeed of cellular GPCRs, in certain biological scenarios.

## 3. US28 Constitutive Signaling during Lytic Infection Activates Multiple Distinct Signaling Pathways, Altering the Infected Host Cell Environment

**Figure 2:**
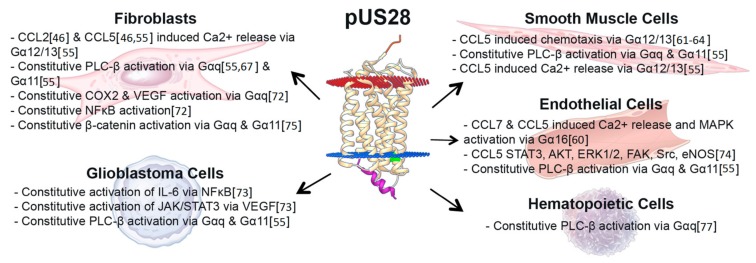
Summary of pUS28-mediated signaling during HCMV infection. pUS28-mediated signaling during infection of the indicated cells. An additional summary of signaling pathways that are modulated in response to pUS28 overexpression is found in Table 2. The pUS28 structure in the center is based on the pUS28 crystal structure, bound to CX_3_CL1 ([34]; PDB ID: 4XT3), using Chimera [35] and the Orientations of Proteins in Membranes (OPM) databases [36].

US28 signaling during lytic infection has been studied extensively, providing us with a broad understanding of how pUS28 signaling affects diverse cell types in assorted ways (Figure 2 and Table 2). The structure of pUS28, and models for GPCR signaling, allow for the possibility that it can spontaneously adopt an open conformation without binding a ligand, thus rendering this viral GPCR constitutively active in some cases [27,30,34].

pUS28 signals constitutively via multiple Gα proteins [65,71,78], resulting in the activation of different signaling pathways. pUS28 binds Gα proteins at the highly conserved “DRY” motif (Figure 1) found in all chemokine receptors [28]. A point mutation in the arginine residue of pUS28’s DRY motif (pUS28-R129A) disrupts and uncouples G protein binding. The net effect of this mutation is a significant reduction of both constitutive and ligand-induced signaling [49,55,70,72]. While the pUS28 DRY motif is necessary for constitutive signaling by this viral GPCR, the pUS28 C-terminus is not required for constitutive signaling [79]. pUS28’s C-terminus is, however, heavily phosphorylated by multiple cellular kinases including GRK2 and GRK5, PKC, and casein kinase-2, which modulate pUS28 signaling, as pUS28’s phosphorylated C-terminus binds β-arrestin, causing internalization and recycling to the plasma membrane [80,81,82,83]. Truncation of pUS28’s C-terminal domain leads to increased and prolonged pUS28 constitutive signaling via PLC-β [84], though no additional phenotypes have been associated with this mutation. The pUS28-R129A mutant, along with other mutants that alter ligand binding to pUS28, has proven vital to our understanding of pUS28 signaling, by allowing us to distinguish pUS28 ligand binding and trafficking from pUS28 signaling [70,77,79,84]. pUS28-R129A has also served as a useful control protein, as it is a “signal-dead” mutant that is nonetheless expressed, controlling for the effects of protein expression on the cell [56].

Although pUS28 is associated with many signaling pathways, some activities have yet to be confirmed during the context of infection (Table 2), while others were confirmed during in vitro infection of cells with HCMV (Figure 2). Nonetheless, the net effect of pUS28-mediated constitutive signaling alters the cellular environment and impacts viral pathogenesis. During lytic infection, pUS28 promotes proliferative signals via PLC, mitogen-activated protein kinase (MAPK), Wnt, and NFκB [55,65,73,75] to influence chemotactic and mitotic processes (discussed below in Section 7). The constitutive signaling activity of pUS28 via the NFκB and MAPK pathways also results in the transactivation of the major immediate early promoter (MIEP), which several groups have shown helps drive lytic HCMV infection [85,86,87]. Thus, HCMV has developed a powerful signaling tool that will function regardless of the presence of specific ligands. Importantly, pUS28 appears to constitutively activate diverse signaling pathways in different cells, and therefore is likely performing specific, assorted functions in a cell-type dependent manner. How or why this occurs remains to be understood. Differences in Gα protein expression between distinct cell types could explain some of these variances, as could the presence of other GPCRs that may interact with pUS28. Alternatively, other subtle cellular differences may potentially affect the open conformation pUS28 adopts. That pUS28 has acquired diverse, tissue-type specific signaling functions could reveal clues as to its role during viral pathogenesis. For example, pUS28-induced migration in smooth muscle cells (SMCs) enhances CCL5-induced chemotaxis [61,64], while pUS28-induced prosurvival signals in glioblastoma multiforme (GBM) cells could favor a more malignant phenotype [70,72,73,74,76], suggesting pUS28-mediated changes in the host cell environment influence disease-specific phenotypes associated with HCMV.

pUS28’s constitutive signaling activity also interferes with the potentiating activity of cellular GPCRs. For example, pUS28 signaling ablates CXCR4 chemokine-induced signaling, as well as membrane trafficking and arrestin binding in HEK293T cells. This signal attenuation is not due to the dimerization of pUS28 with CXCR4, but is rather a result of signaling cross-talk, as attenuation of CXCR4 signaling is lost when pUS28’s DRY motif is disrupted (R129Q) [88]. These findings suggest that pUS28 constitutive signaling causes cross or heterologous desensitization of receptors that is likely due to the constitutive activation of PLC.

pUS28 is additionally implicated in the HCMV-mediated restructuring of lipid rafts in fibroblasts, by enhancing cholesterol efflux from HCMV infected cells [89]. This restructuring of lipid rafts is associated with impaired immune-related signaling by other pathogens, and consistent with this, inflammatory cytokine secretion by mouse macrophages (RAW264.7 cells) was impaired when pUS28 was expressed in isolation. This phenomenon describes a novel role for pUS28 during HCMV lytic infection, as an immune evasion molecule which impairs the host cell innate immune response to HCMV infection [89]. Continued work aimed at unraveling the role pUS28 plays in various tissues and how it interacts with viral and cellular GPCRs will undoubtedly provide us with an enhanced understanding HCMV pathogenesis.

## 4. Chemokine Ligand-Induced pUS28-Mediated Signaling during Lytic Infection

Upon ligand binding at a GPCR’s N-terminus, the chemokine subsequently embeds within the transmembrane domains to stabilize the active conformation of the receptor. pUS28 is a unique GPCR, in that it not only signals constitutively, but also signals in response to ligand binding (Figure 2 and Table 2). pUS28 displays homology to cellular CCR1 and CCR5 [59], as well as CX_3_CR1 [90]. In turn, pUS28 has retained the ability to bind the natural ligands for all these receptors: CCL1, CCL2, CCL3, CCL4, CCL5, and CX_3_CL1 [59,65,69,90,91]. Unusual for a chemokine receptor, this signifies that pUS28 binds both CC and CX_3_C chemokines, but there are no reports to date detailing pUS28’s interaction with any known member of the CXC-chemokine family. pUS28 binding of CC and CX_3_C chemokines is mediated by both the N-terminal domain of pUS28, which contains the chemokine binding site, TTEFDY, as well as pUS28’s transmembrane helices, which form a deep pocket into which the chemokine binds to activate G-protein signaling [34].

Recent work from the Garcia Lab has resulted in the visualization of the interaction between CX_3_CL1 and pUS28 by X-ray crystallography by inducing random mutations into the CX_3_CL1 amino terminus [92]. Calcium release studies using these CX_3_CL1 mutants show that pUS28 remains activated by CX_3_CL1, despite these significant structural differences [92]. Crystallographic analyses of the interaction between pUS28 and CX_3_CL1 reveals that the mutant binds this viral receptor, but is rotated 16.8° and contacts the receptor via different amino acids when compared to the wild type ligand. This is the first investigation to provide a structural understanding for pUS28’s promiscuous ligand binding, suggesting that pUS28 is forced open by ligand binding, rather than activated by specific amino acid contacts [92]. In the future, it would prove interesting to compare the differences between the pUS28-CX_3_CL1 interaction that of an additional ligand, such as CCL5. How these ligands differentially contact pUS28 could explain this viral GPCR’s diverse signaling properties in varied cell types.

In agreement with structural investigations of pUS28, genetic manipulation of *US28* has shown that mutation of its N-terminal chemokine binding site greatly reduces the affinity of pUS28 for all chemokines, and while the aforementioned recent studies have given us greater insight into pUS28-CX_3_CL1 binding, it is clear that pUS28 binds the CC and CX_3_C chemokines differently. In fact, CCL5 and CX_3_CL1 binding are disrupted by mutation of distinct residues [93], suggesting that each of these chemokines interact with specific amino acids within pUS28’s N-terminus. The fact that pUS28 binds CC chemokines and CX_3_CL1 differentially suggests that it may adopt diverse open conformations for distinct ligands, which is consistent with previous findings with regards to other GPCRs [22,23,24]. This concept helps us to understand the observation that these discrete chemokines, upon binding to pUS28, mediate various signaling functions, which ultimately alter the host cell environment [62,64].

Ligand-induced pUS28-mediated signaling results in the release of intracellular calcium, activation of the small G-protein, Rho, and activation of the Focal Adhesion Kinase (FAK) when US28 is expressed in isolation in SMCs and macrophages [62,64]. This demonstrates that upon ligand binding, pUS28 likely adopts an open conformation that activates a different subset of the pUS28-driven signaling pathways from those through which it signals constitutively, as described above. For example, pUS28-mediated calcium release, typically regulated through Gα_q_-PLC-β signaling, is dependent on CCL5 or CX_3_CL1 ligand binding in fibroblasts (human foreskin fibroblasts; HFFs) [46,55,60]. However, PLC-β is also constitutively activated by pUS28 in the same cell type, but calcium is not subsequently released [55,67,84]. This suggests that pUS28 expression in HFFs constitutively adopts an open conformation that activates PLC-β via Gα_q_. There are possible ways to explain why pUS28 alters its behavior upon ligand binding. For example, upon binding either CCL5 or CX_3_CL1, pUS28 adopts an open conformation, perhaps distinct from that which it constitutively adopts, to also activate calcium release via Gα_q_ in HFFs and human coronary artery smooth muscle cells (HASMCs). Alternatively, it is possible that a higher percentage of the pUS28 receptors adopt this open conformation in these cells, enabling the detection of a rapid, short-lived calcium spike. In U373MG (a glioblastoma cell line), however, pUS28 potently activates PLC-β in a constitutive manner, but fails to activate calcium in response to a ligand [55], perhaps suggesting that the number of receptors that adopt this conformation in these cells is reduced. Hence, while pUS28 activates PLC-β across cell types, the downstream consequences are not identical. This variance in ligand-dependent calcium activation between these two cell lines could be due to availability of Gα_q_, which mediates this signaling. Alternatively, perhaps pUS28 fails to adopt the same open conformation in U373MG cells, thus Gα_q_ cannot couple upon ligand binding, a concept that warrants further investigation. In Cos-7 cells, pUS28 constitutively activates PLC-β via Gα_q_, which is unaffected by the addition of CCL5, but is attenuated by CX_3_CL1 binding [65]. This data suggest that when transiently expressed in Cos-7 cells, pUS28 adopts one open conformation to activate Gα_q_, but it displays a mutually exclusive conformation that is dependent upon CX_3_CL1 binding. Alternatively, transient expression may not wholly recapitulate pUS28’s functional capabilities when it is expressed during infection, and indeed, this effect is Cos-7 cell-specific [92]. Nonetheless, taken together, these findings highlight pUS28’s multifunctional signaling properties that vary radically between cell types.

Another demonstration of pUS28’s varied signaling activity is in its “functional selectivity” in mediating chemotaxis [30,94]. CC-chemokines bind pUS28 to promote the migration of SMCs via Gα_i/o_, Gα_16_, and Gα_12/13_, but CX_3_CL1 binding to pUS28 blocks this migration by promoting pUS28 coupling to Gα_q_. In contrast, CX_3_CL1 binds pUS28 to induce macrophage migration, while the CC-chemokine, RANTES (or CCL5), acts as a competitive inhibitor of this process [30,61,64]. Given their competitive effects, it is not surprising that CC-chemokines and CX_3_CL1 differentially interact with the N-terminus of pUS28, as detailed above [93]. Hypothetically, the ability of various ligands to bind pUS28 could stabilize distinct open confirmations, leading to differential binding of Gα proteins. While these data certainly give insight into pUS28-mediated chemotaxis in cells that express this viral GPCR in isolation, additional experiments that confirm these findings in the context of infection are warranted to fully understand pUS28’s role in this cellular process. Nonetheless, the data highlight that mechanisms involved in ligand-regulation of pUS28 signaling remain poorly understood.

## 5. US28 Localization during Lytic Infection

pUS28 is expressed during lytic infection with early kinetics [46,95] and when expressed in isolation, is localized mainly to endocytic vesicles, with only around 20% of the protein localized to the plasma membrane [66]. At late times post-infection, pUS28 concentrates to the viral assembly complex, a large perinuclear structure [48]. Hence, it is not surprising that pUS28 is detected in the mature HCMV virion [52], along with other GPCRs [40,45,47,96]. 

Apart from chemokine binding, the N-terminal region of pUS28 is also involved in protein trafficking, as mutation of tyrosine-16 to alanine (Y16A) abrogates pUS28 trafficking to the plasma membrane [93]. Some of the trafficking functions of the pUS28 N-terminus are perhaps explained by its post-translational modifications, including putative sulfonation at tyrosine-16 and *O*-glycosylation of threonine-12 [93,97]. Despite some speculation, no *N*-glycosylation has been detected to date in the amino acid 30-32 region of pUS28, though there is a putative *N*-glycosylation site 14 amino acids downstream of the TTEFDY chemokine binding site [48].

Like other GPCRs, ligand binding to pUS28 at the plasma membrane causes internalization of the receptor via phosphorylation of its C-terminus and arrestin binding, which likely increases receptor recycling. This causes temporary signal deactivation and is followed by recycling pUS28 back to the plasma membrane [66,98]. This recycling of pUS28 binds and removes chemokines from the environment around HCMV infected fibroblasts [98,99]. Thus, pUS28 creates a “chemokine sink”, as it functions to sequester chemokines from the cellular environment, effectively reducing chemotactic attraction of immune cells to HCMV lytically infected cells in vitro [99]. The biological relevance of this "chemokine sink", however, is challenged by pUS28 overexpression in endothelial cells, which fails to prevent chemokine-induced monocyte adhesion. This suggests pUS28 is unable to overcome the abundant chemokines, likely present in high physiological concentrations [100]. It is also possible, however, that pUS28 displays this function during latent infection, perhaps “mopping up” pro-inflammatory chemokines to facilitate immune evasion by latently infected cells, discussed in detail below.

## 6. The Roles of pUS28 in Latent Infection

Herpesvirus latent infection is defined as the maintenance of the viral genome, coupled with the absence of infectious virus production, with the potential to reactivate in response to the proper cues [101]. Viral latency underpins HCMV persistence by evading detection by the robust anti-HCMV immune response in healthy individuals [102]. As HCMV is a serious threat to immunocompromised and immunosuppressed patients, such as transplant recipients, understanding HCMV latency is vital to preventing disease in these patients. HCMV latency is associated with the expression of a subset of HCMV genes, of which *US28* is one. *US28* mRNA was first detected in latently infected THP-1 cells [103], and later by gene array analyses following infection of primary ex vivo cultured CD34^+^ HPCs [53,104,105], as well as naturally latently infected peripheral blood monocytes [106] and CD34^+^ HPCs [53]. Although *US28* mRNA was not detected by one RNA-seq screen of latently infected CD14^+^ monocytes [107], a more recent RNA-seq analysis detected *US28*, as well as *UL33* and *UL78* mRNA, during experimental and natural infection of CD34^+^ HPCs [53]. This was the first report detailing the latency-associated expression of the other GPCRs, *UL33*, and *UL78*, and these findings confirm previous work from our lab that revealed *US27* is not detectable during experimental latency [52]. The biological role(s) that pUL33 and pUL78 play during latency and reactivation is still unknown, though it is attractive to speculate that their function(s) may prove important to these phases of infection. Indeed, M33 is important for murine cytomegalovirus (MCMV) latency, as the gene is required for efficient establishment of latency in the spleen and lung [108], as well as persistent replication in the salivary gland [109], while deletion of the rat CMV (RCMV) ortholog, R78 attenuates this virus [110].

**Figure 3:**
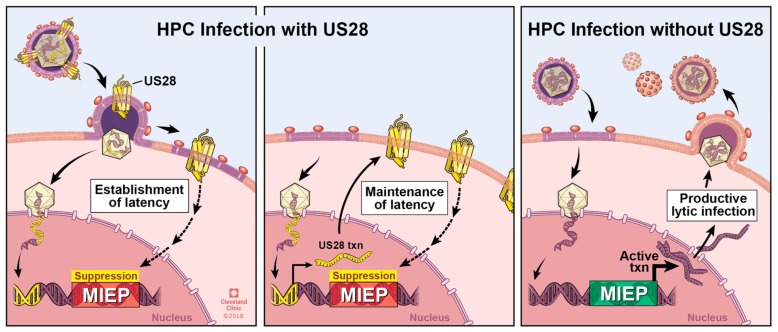
pUS28 contributes to latent infections. Left & Middle Panels: WT infection of HPCs results in a latent infection. Left Panel: pUS28 (yellow barrels) is incorporated into the virion and is delivered upon infection [52]. Virion-delivered pUS28 aids in suppressing transcription (txn) from the MIEP [111,113], by an as of yet unidentified mechanism (dashed arrows). Middle panel: Sustained pUS28 expression throughout infection of HPCs is required for continued suppression of the MIEP, due at least in part through attenuation of MAPK and NFκB (dashed line; [56]). Right panel: When the ORF encoding *US28* is deleted from the viral genome, infection of HPCs results in a lytic, rather than latent infection [52].

*US28* expression in early myeloid lineage cells is required to maintain latent infection (Figure 3) [52,56]. Ectopic expression of a pUS28 R129A signaling mutant in THP-1 cells fails to suppress the MIEP-driven transcription compared to wild type pUS28 ectopic expression [56]. Consistent with this finding, we have shown that the R129A mutant in the context of infection fails to maintain latency in Kasumi-3 cells or primary CD34^+^ HPCs [111]. Together, these data argue a role for pUS28-mediated signaling in maintaining a latent infection. However, a pUS28 Y16F mutant, which abrogates CCL3, CCL4, CCL5, and to a lesser, though significant extent, CX_3_CL1 binding [93], can maintain latency [56], suggesting that pUS28’s ability to influence latency through its signaling abilities is independent from binding these particular ligands. While the nuances of pUS28-directed signaling during latency are far from understood, this viral GPCR appears to act on multiple signaling pathways during expression in or infection of cells that support HCMV latency. Ectopic expression of pUS28 in THP-1 cells results in PLC-β activation, and importantly, this finding was confirmed in the context of infection, by comparing PLC-β activity in wild type and *US28*-deletion virus infected THP-1 cells [77], the latter of which is an observation that is consistent with findings in fibroblast cells [84]. Furthermore, this PLC-β activation promotes THP-1 cell adhesion to endothelial cells [77], a finding that suggests pUS28 may function to promote monocyte–endothelial cell interaction during the context of disease, discussed in detail below in Section 8. Additionally, pUS28 also activates the signal transducer and activator of transcription 3-inducible nitric oxide synthase (STAT3-iNOS) signaling pathway in CD34^+^ HPCs [112], consistent with findings in GBM cells [73]. The result of this activity serves to reprogram these CD34^+^ HPCs into a subset of immunosuppressive monocytes, which fails to occur following infection with a *US28*-deletion virus [112].

Perhaps the most surprising observation thus far is that pUS28 attenuates at least two signaling pathways during latency, which it otherwise activates during lytic infection [56]. This study revealed that pUS28 attenuates MAPK and NFκB in latently infected THP-1 cells. Furthermore, MIEP-driven transcription was attenuated as a result [56], suggesting that pUS28 alters signaling to silence this very strong viral promoter. Differentiation of THP-1 cells following phorbol ester treatment, leading to viral reactivation, revealed that pUS28 indeed no longer suppressed but rather activated these pathways, as well as MIEP transcription [56]. This is perhaps the starkest observation that pUS28 shifts its function in a cell-type dependent manner, and in a way that supports either HCMV latency or viral reactivation. The molecular mechanism by which pUS28 can achieve this remains unclear, but it is not unreasonable to speculate that pUS28 may couple to different Gα proteins before and after cellular differentiation. Other differentiation-specific vicissitudes in the cellular environment, such as altered viral and cellular chemokine expression, and/or post-translational modifications of pUS28, may certainly also contribute pUS28’s varied functions during latency, reactivation, and subsequent lytic infection.

Many questions remain regarding pUS28’s role during latency. Most recently, our results show that pUS28 is required for both the establishment and maintenance of HCMV latency (refs. [111,113], Figure 3). To maintain HCMV latency, it seems very likely that pUS28’s signaling capabilities are required, however the specific cellular signaling pathways that pUS28 modifies during this phase of infection remain unknown. These certainly will include the activation or attenuation of specific pathways, as mentioned above, though our current knowledge is likely far from complete, given the multitude of signaling pathways associated with pUS28 during the lytic lifecycle. With the multifunctional nature of pUS28 signaling during lytic infection, it is not surprising to find that pUS28 both manipulates the cellular environment to repress the MIEP, thereby maintaining latent infection, while also directing the cell towards monocyte subsets that support latent infection. There is a growing body of literature that continues to reveal that HCMV latent infection has many different phenotypic effects on the latently infected cell, thus further research is quite likely to uncover additional key roles for pUS28 during this phase of viral infection.

## 7. US28 and Cancers

HCMV infection is associated with a number of cancers including prostate cancer, colon cancer, alveolar soft part sarcoma, Epstein Barr virus (EBV)-negative Hodgkin lymphoma, and gliomas [114,115,116,117,118,119,120,121]. While there is no evidence that HCMV is the etiologic agent of any cancer, it is considered by many as an oncomodulatory virus, as it interacts with various cellular signaling pathways that could promote a malignant phenotype [122]. This is an important distinction from oncogenic viruses, such as Kaposi’s sarcoma-associated herpesvirus (KSHV) or EBV, which are indeed the cause of Kaposi’s sarcoma (KS), primary effusion lymphoma (PEL), and multicentric Castleman’s disease (MCD) or Hodgkin lymphoma, naso-pharyngeal cancer, and Burkitt lymphoma, respectively [123]. Work from a variety of labs support the link between HCMV and GBM, a highly aggressive glioma of astrocytic origin [124,125], where the mean two-year survival is 15–25%. HCMV DNA and protein were detected in glioblastoma biopsies [117,118,119], and higher grade HCMV infection correlates with poorer survival of GBM patients [126]. Targeting HCMV has also showed promise for GBM patients, who when treated with valganciclovir in addition to standard care had an improved two year survival rate of 62% compared to 18% for the control group, in one prospective study [127] and median overall survival increased from 8.7 to 13.1 months in another study, where valganciclovir was provided with the antiangiogenic antibody, bevacizumab [128]. It is important to note that each of these represent small, single trials, and thus further studies aimed at determining the significance and the impact of such treatments are warranted. The link between HCMV and various cancers, including GBM, remains controversial, as studies from various groups find contradicting or inconclusive evidence. For example, some studies have failed to detect CMV DNA in glioblastoma tissue [129,130,131], while another group was unable to detect CMV RNA [132], suggesting that CMV does not replicate in GBMs, or paradoxically, that CMV proteins are present in GBMs in the absence of detectable viral DNA/RNA [133]. In another study, only half of patients with gliomas that were positive for CMV DNA had detectable CMV DNA in their blood [134]. Others have found that the platelet-derived growth factor receptor (PDGFR), which HCMV uses to infect epithelial, endothelial, and fibroblast cells [135], displays increased expression in patient gliomas compared to normal tissue [136], leaving the possibility that glioma formation may promote CMV infection and not the other way around [137]. Epidemiological studies have also failed to find a link between CMV and gliomas [137]. Indeed, one study treating GBM with valganciclovir as an add-on therapy had disappointing results [138]. Links between CMV and other cancers, such as medulloblastoma [139], breast cancer, have met with similar problems [140]. Overall, the potential for HCMV’s actions as an oncomodulatory virus seem reasonable, however the extent to which HCMV is present in cancers, whether the virus replicates in cells found in the tumor and the degree of the virus’s contribution to cancer progression are far from clear.

Investigators have focused on pUS28 as a prime candidate that could regulate, at least in part, HCMV’s proposed oncomodulatory effects in the tumor environment. pUS28 activates a variety of signaling cascades that can function to promote cancer, including PLC [55], cyclooxygenase 2 (COX2) via NFκB [70,72], Wnt [75], hypoxia-inducible factor 1α (HIF1α) [76], and the STAT3-interleukin 6 (IL6) axis [73,74]. Indeed, pUS28 expression in isolation promotes tumorigenesis in mouse models of GBM via these pathways [70,72,141]. Importantly, pUS28 is detected in patient GBM specimens [73,141,142,143] including one study in which US28 mRNA was present in 67% of tested patient GBM samples. In this same study, the investigators found that pUS28 promoted STAT3-eNOS mediated secretion of vascular endothelial growth factor (VEGF), as well as activated extracellular-signal-regulated kinase 1/2 (ERK1/2), FAK, and Src in neural precursor cells from hippocampus tissue [74]. All of these pathways are associated with proliferative and prosurvival phenotypes critical to the progression of tumors, particularly STAT3 [144,145]. Indeed, STAT3 mediated secretion of IL6 would promote tumor growth and survival, in a positive feed-forward loop [146,147]. As such, in a Mut3 transgenic mouse model (a PTEN haploinsufficient mouse line with accelerated astrocytomas), MCMV infection resulted in STAT3 activation and also significantly shortened the lifespan of mice with GBM [148]. Using a recently developed nanobody against pUS28, investigators have shown reduced pUS28-induced secretion of VEGF from U251 cells (a GBM cell line), which subsequently decreased pUS28-induced spheroid formation (discussed in further detail below) [141]. Thus, there is clear evidence that pUS28 activates pathways in neuronal cell lines in such a manner that promote proliferative and prosurvival signals that could exacerbate GBMs. Continued efforts towards understanding HCMV, and specifically pUS28, during gliomas will reveal the contributions of infection toward glioma progression.

In addition to its association with GBM, pUS28 is also linked to the progression of other cancers. pUS28 expression also promotes intestinal neoplasia in transgenic mice [149] and may prove a prognostic of poor survival in colorectal cancer [150]. Taken together, these findings demonstrate that pUS28 signaling has the potential to exacerbate malignant phenotypes in HCMV-positive patients with cancer. Overall, there is now a consensus among some researchers that HCMV plays an oncomodulatory role in some cancers, such as glioblastomas [122], but the extent to which HCMV contributes to other cancers and neoplasia initiation remains to be elucidated. Given the extent to which pUS28 is a potent, pro-inflammatory signal activator in many models of lytic infection, it is likely that if HCMV proves to be oncomodulatory, then pUS28 itself may prove a key factor in linking HCMV to cancers.

## 8. US28 in Vascular Disease

HCMV infection is associated with a significantly increased relative risk of CVD, including atherosclerosis, restenosis following angioplasty, and transplant-associated vasculopathy in solid organ transplant patients [151,152], which was recently confirmed in a meta-analysis of prospective studies [152]. Atherosclerosis is a common CVD that has a complex and multifactorial etiology, characterized by endothelial dysfunction, monocyte recruitment, increased SMC proliferation and migration, and infiltration of macrophages to the arterial wall. Additionally, inflammation of the vasculature leads to an increase in chemokines, cytokines, and adhesion molecules, which in turn mediate, the aggregation and/or adhesion of inflammatory cells to the vascular walls [153]. These processes promote plaque formation, which once destabilized and mobilized, can result in thrombosis, myocardial infarction, and/or stroke [154].

**Figure 4:**
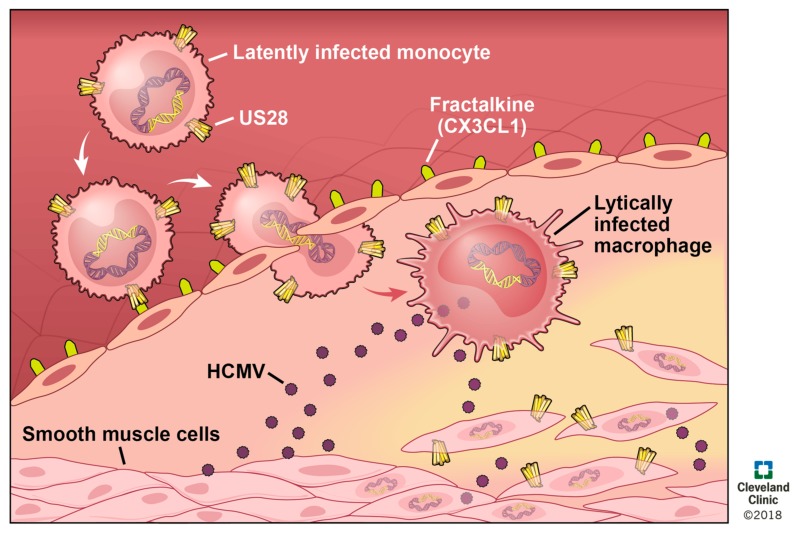
A model of pUS28’s role during atherosclerotic plaque formation. Monocytes are recruited to the injured endothelium, after which they extravasate through the endothelium into the intima, resulting in their differentiation to macrophages. SMCs then migrate towards the site of the developing plaque in response to pro-inflammatory signals. pUS28 expressed from latently infected monocytes can interact with fractalkine, upregulated on the endothelium, thus enhancing the binding of these cells. The differentiation of these cells to macrophages will result in lytic replication, capable infecting neighboring cells (e.g., macrophages, endothelial cells, and SMCs). Infection of SMCs leads to pUS28 expression, which is sufficient to induce the migration of this cell type [62]. Together, it is possible that HCMV, and specifically pUS28, could accelerate the cellular processes that lead to plaque formation.

HCMV seropositivity is linked to a higher rate of mortality from atherosclerosis [155,156], and infection with MCMV increases the severity of atherosclerosis in the *apoE^−/−^* murine model of the disease. HCMV DNA is detected in diseased vessels and in SMCs in early stage atherosclerotic lesions (albeit also in nondiseased SMCs) [157,158]. Viral infection of the vascular endothelium creates a pro-inflammatory environment, and increases both adhesion properties and permeability of the endothelium [159]. Thus, it is attractive to speculate that circulating, infected monocytes could adhere, extravasate, and then differentiate into macrophages, which would result in HCMV reactivation (Figure 4). This could then disseminate lytically replicating virus to other cells of the vasculature, including SMCs. In summary, HCMV infects and modulates cell types involved in atherosclerosis, and together support a significant biological role for HCMV in atherosclerotic progression, yet how this occurs is not known.

It is attractive to speculate that pUS28 perhaps plays a role in this HCMV-mediated progression of atherosclerosis (Figure 4). For instance, ectopic pUS28 expression induces chemotaxis in the presence of CC-chemokines in SMCs as well as macrophage chemotaxis in the presence of CX_3_CL1 [64]. pUS28 could, therefore, contribute to atherosclerotic disease by promoting the migration to and subsequent accumulation of SMCs at the site of plaque formation, as well as the continued infiltration of inflammatory macrophages into this inflamed milieu. pUS28 also induces the release of pro-inflammatory [55,70,75] and pro-angiogenic cytokines [160], which contribute to chemotaxis [64], cell survival, and the pro-inflammatory [72,73,149,160] feedback loop that exacerbates atherosclerosis. Finally, pUS28 expression in the monocytic THP-1 cell line resulted in increased adhesion to human vascular endothelial cells (HVECs). This adhesion is mediated by constitutive signaling, while the N-terminus of this viral receptor is not required to see this effect [77]. These data suggest that pUS28 expression during latency in monocytes may also promote monocyte adhesion to the site of atherosclerotic plaque formation (Figure 4), which is clearly of etiological importance. The environment of these plaques are already pro-inflammatory in nature, and coupled with the fact that monocytes differentiate to macrophages in parallel with extravasation into the intima, it is attractive to reason that an infected monocyte will thus reactivate HCMV upon differentiation [161,162,163], thereby creating a positive feedback mechanism that accelerates the cellular processes that dictate atherosclerotic plaque formation. Overall, the link between HCMV and CVD is well-supported, and pUS28 plays a key role in the cellular processes that contribute to such diseases, both by augmenting the inflammatory nature of CVDs, as well as more specifically promoting the invasive and adhesive phenotypes of the diseased cells found in atherosclerotic plaques. Additional work aimed at completely understanding the impact of pUS28 on atherosclerosis and other CVDs could prove significant in developing novel therapeutics, which could significantly reduce, or at least delay, CVD incidence. A US28 inhibitor, either acting as an antagonist to CC-chemokine and CX_3_CR1 binding to pUS28 to block chemotaxis, or a full inverse agonist, which could also reduce pUS28-mediated inflammatory activation, could have significant clinical benefits for this patient population.

## 9. US28 as a Therapeutic Target

As mentioned above, *US28* is dispensable for HCMV lytic infection in fibroblasts [46], but does play an augmenting role during epithelial cell infection [48]. Targeting pUS28 as an antiviral against lytic infection may therefore prove less effective. However, as pUS28 is associated with pro-inflammatory and proliferative phenotypes, targeting pUS28 could serve as an additional effective treatment against CVD and tumor growth [147,164]. 

The basal activity of a GPCR is altered by the addition of different types of agonists. A GPCR agonist (full or partial agonist) is defined as a ligand or compound that increases the signaling from the receptor above basal levels after binding. An inverse agonist, therefore, is a protein, molecule, or compound that opposes the agonist or active state of the receptor, thus inverse agonist binding to a GPCR reduces its basal signaling. Finally, an antagonist is a ligand or molecule that binds a GPCR, but does not alter the receptor’s balance between its active and inactive states. Several groups have taken aim at pUS28 signaling to develop compounds that function as antagonists or inverse agonists. However, inhibiting pUS28 has proven complicated due to its multifaceted signaling properties. 

pUS28 antagonists, starting with methiothepin and later octoclothepin [165], were designed to block CCL5 binding to pUS28, but they both also partially inhibit constitutive signaling [147,165]. Thus, these compounds are not true “antagonists” by definition, but rather are partial inverse agonists. These compounds could reduce pUS28 constitutive signaling perhaps by tipping the balance towards trafficking the receptor to lysosomes, rather than receptor recycling, therefore appearing to act as inverse agonists. Other investigators have targeted pUS28’s constitutive signaling properties using compounds, such as the CCR1 antagonist, VUF2274, which lowers the constitutive signaling activity of pUS28 while also sterically blocking chemokine binding [51,166]. While VUF2274 is a compound that appears to function as an inverse agonist vis-à-vis PLC signaling, this remains undetermined. The effect of VUF2274 treatment on receptor recycling likely has a significant impact on the constitutive signaling of the receptor. Hence, until such details are revealed, we encourage caution in referring to compounds such as VUF2274 as inverse agonists until they are indeed proven to function in this manner. Nonetheless, it is interesting to note that VUF2274 can block chemokine binding, as it binds pUS28Δ2-22, an amino-terminal truncated mutant, which has abrogated chemokine binding. This shows that VUF2274 must bind pUS28 away from the N-terminus, yet it can still compete with chemokines for binding [51]. This is similar to how this compound functions as a CCR1 antagonist, however, upon binding pUS28, VUF2274 may also force pUS28 into a closed conformation in order to act as an inverse agonist. Recently, VUF2274 was used as an inverse agonist of pUS28 in order to inhibit the maintenance of HCMV latent infection. VUF2274 treatment of latently infected cells forced HCMV into lytic infection and resulted in the presentation of HCMV antigens on infected monocytes, allowing for their detection and killing by HCMV-specific T cells isolated from HCMV seropositive donors [56]. Although VUF2274 showed significant toxicity towards uninfected ex vivo cultured monocytes, this is proof of principle that inhibiting pUS28 with this inverse agonist, coupled with standard antiviral therapies that eliminate lytic infections, could constitute a therapy to kill cells latently infected with HCMV. Moving forward, modifications were made to VUF2274, which have improved its affinity to pUS28 [167,168]. Additional pUS28 inverse agonists have been developed, based on hydroisoquinoline antagonists of CX_3_CR1 [169], flavonoids [170], or biphenyl amides [171]. Continuing these efforts, investigators have shown that C-terminal truncation of pUS28 (pUS28Δ300) is an effective tool for screening potential inhibitors, as it has reduced membrane recycling, which otherwise camouflages the agonism of some ligands towards pUS28. This mutant was used to discover new scaffolds that could lead to the development of novel pUS28 inhibitors [172,173]. Such studies show promise in the expansion of our arsenal of pUS28 inhibitors that are more effective than VUF2274. Indeed, further reduction in cytotoxicity could render these compounds suitable for clinical applications. Such compounds could act as inverse inhibitors against pUS28 as a “kick and kill” therapy, whereby HCMV reactivation is triggered, making the reactivated cells susceptible to immune recognition [56]. One could envision providing such a therapy to an organ donor, prior to transplant to purge the infected cells, which would otherwise transfer to the immunosuppressed recipient during transplant, which often leads to devastating CMV disease. 

In addition to small molecule inhibitors, others have targeted pUS28 with macromolecules. Heukers et al. have isolated a bivalent nanobody based on its ability to displace CCL5 binding to pUS28. Promisingly, this nanobody appears to act as an inverse agonist, preventing constitutive pUS28 activation of NFκB and inositol triphosphate (IP_3_) accumulation. Furthermore, this nanobody impairs *US28*-enhanced growth of U251 cells, a GBM cell line, as well as HCMV-infected primary GBM cells [141]. The production of a nanobody that acts as an inverse agonist is particularly interesting, as it perhaps binds pUS28 in a manner that closes pUS28’s open conformation. Much of the structural work aimed at understanding the relationship between GPCRs and the ligands and compounds with which they interact suggest a “conformational selection” model, such that the ligand binds to and stabilizes a particular conformation. In this case, the nanobody may preferentially bind to pUS28’s closed conformation, thereby stabilizing it in the closed, rather than open, conformation. The potential applications of this nanobody in the treatment of GBM remain unclear, however, and depend entirely on the extent to which HCMV modulates GBM and the mechanisms underlying pUS28’s contribution to HCMV oncomodulation. These questions remain unanswered, as different studies have suggested widely different extents to which HCMV contributes to GBM formation and progression, as discussed above in Section 7. Perhaps an additional use of this anti-US28 bivalent nanobody would include the treatment of latently infected individuals. As discussed above, the pUS28 inverse agonist, VUF2274 induces viral reactivation in latently infected monocytes. Thus, this nanobody could function similarly, but without the additional toxicity associated with VUF2274 [56]. 

Another effort to target pUS28 with a macromolecule details the use of a fusion toxin protein (FTP), wherein the soluble extracellular domain of CX_3_CL1 was fused to pseudomonas exotoxin A (PE). This FTP exploits the high affinity binding of pUS28 to CX_3_CL1 to target PE to infected cells [174]. An F49A point mutation in the CX_3_CL1 portion of the FTP greatly reduces its ability to bind CX_3_CR1, thereby generating high selectivity towards pUS28 and HCMV infected cells. This therapy, F49A-FTP, showed great potential to target and kill HCMV lytically infected MRC-5 (embryonic lung fibroblast) cells, as well as HCMV infected engraftments in a SCID-hu mouse model of systemic HCMV infection [174]. In addition to models of lytic infection, F49A-FTP was effective at targeting and killing HCMV infected ex vivo cultured monocytes and CD34^+^ HPCs, as well as naturally latently infected monocytes, via pUS28 expressed on the cell surface of these latently infected cells [175]. Exploiting pUS28 expression during latent infection is particularly promising, given the lack of anti-HCMV therapies targeting latently infected cells. F49A-FTP is particularly interesting, as its mechanism of action is effective against both lytic and latent HCMV. pUS28 makes for an attractive drug target, given its cell surface localization even during latent infection [152]. Indeed the findings from this latter study highlight the ability to perhaps eliminate HCMV latently infected cells, thereby preventing viral reactivation. This would serve as a significant advancement in treating patients in the clinic. One could envisage, for example, F49A-FTP treatment of transplanted tissue prior to hematopoietic stem cell transplant to clear latently infected cells. Additionally, this treatment could apply to solid organ transplant patients as well, as a means by which to reduce the risk of post-transplant CMV disease. Further developing F49A-FTP to improve selectivity for pUS28 over CX_3_CR1 is therefore a key priority, given that the binding of CX_3_CR1, which is also expressed in many uninfected cells including monocytes [176], proved the limiting factor in the use of F49A-FTP against latently infected cells [175].

As a GPCR, clearly pUS28 is an attractive target for novel therapeutics. Further dissecting the specific pUS28-manipulated pathways during both lytic and latent infections, as well as continued understanding of pUS28’s structural elements, will prove paramount to rational drug design. Whether these findings result in the development of new compounds or the repurposing of currently approved compounds, new means by which to prevent CMV disease will benefit immunosuppressed individuals, including transplant patients, and perhaps those suffering from CVD or cancer.

## 10. Concluding Remarks

Despite being only one of over 200 ORFs encoded by HCMV, *US28* has garnered much attention from GPCR biologists and HCMV virologists, alike. Much of the large body of literature focused on pUS28 has detailed the multifaceted nature of this viral protein, a chemokine receptor homologue that binds multiple chemokines from both CC and CX_3_C classes that signals both in constitutive and ligand-dependent fashions, resulting in diverse downstream consequences that have proved variable between different cell types. Continued work aimed at understanding this signaling protein in disease, first in atherosclerosis and then in cancer, has provided more details into how pUS28 impacts the host cell environment and viral pathogenesis. Our recent findings detailing the requirement for pUS28 during latency has also opened a new avenue for interrogating the biological functions of pUS28, which we and others continue to pursue. Together, research on pUS28 continues to increase in intensity. It is critical as research on this viral GPCR moves forward that we cross-validate and confirm each other’s findings using parallel, yet slightly different systems, which may allow us to dissect the fine details underlying the relevant biological functions of pUS28. This would undoubtedly propel the entire pUS28 field forward. Uncovering such detail will afford us, as a field, the ability to focus future research that may include using pUS28 as a therapeutic target in some disease settings, while perhaps exploiting it as a biomarker for another. Nonetheless, we will undoubtedly continue to learn more about this multifaceted protein.

## Figures and Tables

**Table 1:** Summary of the HCMV-encoded GPCR functions

HCMV-Encoded GPCR	Function in Lytic Infection of Fibroblasts	Function in Lytic Infection of Other Cells	Function in Latency	Interactions with Other GPCRS
US27	Extracellular virus spread [47]	Dispensable for viral growth in epithelial cells; required for extracellular spread in endothelial cells [47]	Not expressed [52,53]	Enhances CXCR4 activation [42,50]. Heteromerizes with pUS28 [54]
US28	Dispensable [46,48,55]	Augments infection of epithelial cells [48]; dispensable for growth in endothelial cells [55]	Required for latency [52,56]	Heteromerizes with pUS27, pUL33, and pUL78. Interaction with either pUL33 or pUL78 abrogates pUS28-mediated NFκB activation [54]
UL33	Dispensable [40]	Unknown	Detected [53]; unknown function	Heteromerizes with pUS28 and abrogates pUS28-mediated NFκB activation [54]. Binds CCR5 or CXCR4 to reduce signaling activity in both [41].
UL78	Dispensable [44,45]	Efficient viral replication in endothelial & epithelial cells. Efficient entry & virion delivery into epithelial cells [45].	Detected [53]; unknown function	Heteromerizes with pUS28 and abrogates pUS28-mediated NFκB activation [54]. Binds CCR5 to increase signaling activity [41]. Binds CXCR4 to reduce signaling activity [41].

**Table 2:** Summary of pUS28-mediated signaling properties. The effects of pUS28 signaling in the presence or absence of a ligand (middle column) are detailed (right column). The cell type used is shown (left column) and the expression system for experiments providing pUS28 in trans are noted. Experiments that detailed the effects of signaling in response to pUS28 overexpression and in the context of infection are denoted by symbols.

Cell System	Ligands	Phenotypic Change
K562 ^⌘^^f^	CCL2 and CCL5	Calcium release [59]
HEK293T ^§^ & infected HUVECs ^g^	CCL7 and CCL5	Calcium release and MAP kinase via Gα_16_ [60]
Infected fibroblast	CCL2 and CCL5	Calcium release [46]
Infected arterial SMCs	CCL5 (inhibited by CX_3_CL1)	Chemotaxis via Gα_12/13_ [61,62,63,64]
Cos-7 cells ^§^	Constitutive, CX_3_CL1 inhibits	PLC, NFκB via Gα_q_ [65,66]
Infected fibroblasts	Constitutive	PLC via Gα_q_ [67]
Cos-7 cells ^§^	Constitutive, antagonized by CCL5	PLC and NFκB via Gα_o_ and Gα_q11_ [68]
HEK293T ^§^	Constitutive	CREB/NFAT ^a^ via Gα_q_ [69]
NIH-3T3 ^⌘^^h^ & infected U373	Constitutive	VEGF ^b^ secretion via Gα_q_ and MAPK [70]
Cos-7 cells ^§^	Constitutive	Serum response factor via Gα_o_ and Gα_q11_ (inhibited by Gα_16_) [71]
Mouse macrophages ^⌘^	CX_3_CL1 (inhibited by CCL5)	Chemotaxis via Gα_q_ [64]
NIH-3T3 ^§^ & infected fibroblasts	Constitutive	COX2 and VEGF via Gα_q_; NFκB [72]
NIH-3T3 ^§^ & HEK293T ^§^; infected U373MG	Constitutive	NFκB induction of IL6, VEGF secretion inducing JAK ^c^/STAT3 [73]
Non-proliferating hippocampal cells ^¶^ & HUVECs ^¶^	CCL5	“Invasive phenotypes” via STAT3, AKT ^d^, ERK1/2, FAK, Src, and eNOS [74]
NIH-3T3 ^§^ & HEK293T ^§^; infected fibroblasts & U373MG	Constitutive	β-catenin via both Gα_12_ and Gα_q_ together [75]
Infected HASMC, U373MG, HFFs, & HUVECs	Constitutive	PLC-β via Gα_q_ and Gα_11_ in all cell types tested [55]
Infected HASMC & HFFs	CCL5	Calcium release via Gα_12/13_ [55]
U251 ^¶^ & NIH-3T3 ^⌘^	Constitutive	VEGF secretion and HIF1-α activation with Akt and PKM2 ^e^ [76]
THP-1 cells ^¶^	Constitutive	PLC-β via Gα_q_ [77]
THP-1 cells ^⌘^	Constitutive	Attenuation of MAPK and NFκB [56]

^⌘^ stable expression; ^§^ transient expression; ^¶^ stable expression and infection; ^a^ CREB/NFAT; cAMP response element binding/nuclear factor of activated T cells; ^b^ VEGF; vascular endothelial growth factor; ^c^ JAK; Janus kinase; ^d^ AKT; alias, protein kinase B (PKB); ^e^ PKM2; pyruvate kinase muscle isozyme M2; ^f^ K562; human bone marrow lymphoblasts; ^g^ HUVECs; human umbilical vein endothelial cells; ^h^ NIH-3T3s; murine embryo fibroblasts.
